# Enhancing uptake of the Canadian Prison Needle Exchange Program using a non-randomized stepped-wedge cluster type 1 hybrid implementation trial: The NEXUS study protocol

**DOI:** 10.1016/j.conctc.2026.101642

**Published:** 2026-04-28

**Authors:** Olivia Price, Frederick L. Altice, Lynn M. Madden, Monica Taljaard, Mark Stoové, Behzad Hajarizadeh, Lise Lafferty, Nadine Kronfli

**Affiliations:** a Centre for Outcomes Research and Evaluation, Research Institute of the McGill University Health Centre, Montreal, QC, Canada; b National Drug and Alcohol Research Centre, UNSW Sydney, Sydney, NSW, Australia; c Department of Internal Medicine, Section of Infectious Diseases, AIDS Program, Yale School of Medicine, New Haven, CT, USA; d APT Foundation, New Haven, CT, USA; e Department of Epidemiology of Microbial Diseases, Yale School of Public Health, New Haven, CT, USA; f Centre of Excellence for Research in AIDS (CERiA), Faculty of Medicine, University of Malaya, Kuala Lumpur, Malaysia; g Ottawa Hospital Research Institute, Ottawa, ON, Canada; h Centre for Motivation and Behaviour Change, University of Bath, Bath, United Kingdom; i Behaviours and Health Risks, Burnet Institute, Melbourne, VIC, Australia; j School of Public Health and Preventive Medicine, Monash University, Melbourne, VIC, Australia; k Australian Research Centre in Sex, Health and Society, La Trobe University, Melbourne, VIC, Australia; l The Kirby Institute, UNSW Sydney, Sydney, NSW, Australia; m Centre for Social Research in Health, UNSW Sydney, Sydney, NSW, Australia; n Department of Medicine, Division of Infectious Diseases and Chronic Viral Illness Service, McGill University, Montreal, QC, Canada

**Keywords:** Prisons, Needle exchange programs, Implementation science, HIV, Hepatitis C, People who inject drugs

## Abstract

**Background:**

Prison needle exchange programs (PNEPs) are evidence-based harm reduction interventions that decrease bloodborne virus transmission by reducing the sharing of injection equipment. Canada introduced PNEPs in nine of its 43 federal prisons in 2018-2019; however, uptake is low. This implementation gap undermines program effectiveness, jeopardising individual- and population-level benefits of the program. This stepped-wedge cluster non-randomised type 1 hybrid implementation trial will evaluate whether the Network for the Improvement of Addiction Treatment (NIATx) bundle of implementation strategies and tools increases PNEP uptake.

**Methods:**

All nine prisons were allocated non-randomly to three sequences; each sequence is activated at six-month intervals. Over 24 months, each prison will use NIATx tools to address site-specific barriers to PNEP determined during preliminary work. Stepped implementation will be evaluated using the Reach, Effectiveness, Adoption, Implementation, Maintenance (RE-AIM) framework of the Practical Robust Implementation and Sustainability Model (PRISM). The primary effectiveness outcome is PNEP uptake (i.e., number of participants enrolled on the program). Secondary effectiveness outcomes include number of kits distributed (evidencing active participation) and individuals tested and diagnosed with bloodborne virus infections, including HIV and hepatitis B and C virus (to assess harm reduction impact). Staff surveys and focus groups during and post-implementation, respectively, will explore individual- and organisational-level factors influencing implementation and maintenance.

**Discussion:**

We anticipate that NIATx tools will improve uptake of PNEPs among people who inject drugs in Canadian federal prisons. Findings are expected to inform scale-up across remaining Canadian prisons, support long-term sustainability, and inform global advocacy for prison needle and syringe programs.

## Highlights

• Uptake of services within the Canadian prison needle exchange program (PNEP) is low.
• This type 1 hybrid implementation trial aims to increase uptake of the PNEP by reducing implementation barriers.
• In a stepped-wedge design, the NIATx blended implementation strategy will be applied in nine prisons.
• The primary effectiveness outcome is PNEP participation, an indicator of program engagement.
• Implementation outcomes will support sustainability and scale-up of the PNEP.

## 1 Introduction

People who inject drugs are at elevated risk of bloodborne virus infections (e.g., HIV, hepatitis C virus [HCV]), primarily through sharing of injection equipment [1]. This population is also disproportionately incarcerated, with lifetime injection drug use prevalence 50 times higher among people in prison and other closed settings than in the general population [2]. While injection frequency typically decreases during incarceration [3], limited access to sterile injection equipment means that the risk of bloodborne virus acquisition per injecting episode is higher in prisons than in the community [4]. Among people who inject drugs, recent incarceration is associated with a higher risk of HIV and HCV acquisition [5], underscoring prisons as key settings for the prevention of bloodborne virus transmission [[6], [7], [8], [9]].

The World Health Organization and the United Nations Office on Drugs and Crime recommend prison needle exchange programs (PNEPs) as essential components of HIV/HCV prevention strategies [6], based on unequivocal findings that they reduce transmission in community settings by reducing reuse of injecting equipment [10,11]. As of 2025, PNEPs were available in only 11 countries globally, often as pilot programs [12], reflecting sub-optimal implementation, reach, and scale-up. Structural barriers including stigma, political opposition, security concerns, failure to protect participant confidentiality, and restrictive policies have limited broader adoption [13]. This has undermined efforts to reduce the HIV and HCV burden among people who inject drugs and to meet HIV and HCV reduction targets set by UNAIDS and the World Health Organization [14,15].

Research on effective implementation is needed to increase PNEP engagement among people who inject drugs in prison and support sustainable, scalable programs. With only Ukraine having introduced PNEPs in the past decade [13], evidence of program effectiveness is needed to inform broader adoption globally [16]. Canada provides a unique setting for this work, as it is one of few countries where PNEPs are implemented. The Canadian PNEP is cost-effective [17], but it is estimated that fewer than 10% of people who inject drugs in prison access it [18]. This implementation gap is driven by multiple, intersecting barriers, involving the individual, staff, the prison environment itself, and enacted policies governing PNEP. Stigma remains a major issue at all implementation levels and is exacerbated by limited program confidentiality, with participation visible to both healthcare and correctional staff [19]. Staff opposition to the program is often shaped in part by safety concerns, including a fear of needlestick injuries, despite no reported incidents since program inception [13]. Public opposition, including from members of the Union of Canadian Correctional Officers, further limits program uptake, while broader zero-tolerance approaches to drug use in prison contradict patient-centred harm reduction principles [[19], [20], [21], [22]].

The **N**eedle **EX**change **U**ptake **S**tudy (NEXUS) is a type 1 hybrid effectiveness-implementation trial that aims to evaluate the PNEP in Canadian federal prisons via a stepped wedge design, while simultaneously examining contextual determinants. We will apply the Network for the Improvement of Addiction Treatment (NIATx) model as a blended implementation strategy to enhance PNEP delivery.

## 2 Methods

### 2.1 Context

Canadian federal prisons are overseen by Correctional Service Canada and house people serving sentences of two years or longer. Prior to the introduction of PNEPs in Canada, research indicated that drugs were readily available in federal prisons and that sharing of needles and other injection equipment was common [23]. Data collected since program implementation suggest that approximately 60% of people who inject drugs in prison share injecting equipment [24]. It is estimated that 0.7% of people incarcerated in Canadian federal prisons are living with HIV, and 22% have evidence of prior or current HCV infection [25].

In response to legal action, Correctional Service Canada introduced PNEPs in nine of its 43 federal prisons in 2018-2019. In line with the 2024-2030 Government of Canada's Sexually Transmitted and Blood-Borne Infection Action Plan [26], PNEPs were expanded to five additional prisons between 2024 and 2025 (C. Gaudet, personal communication, 18 September 2025). The program is intended to reduce sharing of injection equipment, transmission of bloodborne viruses, and injecting-related skin infections [27]. The PNEP sits within Correctional Service Canada's broader harm reduction program that includes opioid agonist therapies, HIV and HCV treatment, and HIV pre-exposure prophylaxis [27]. Participation in the PNEP is contingent on a ‘Threat Risk Assessment’, a mandatory security approval process unique to Canada's healthcare delivery model [20]. To date, uptake of the PNEP by people who inject drugs in Canadian federal prisons is estimated as less than 10% of those who might benefit from it [18], reflecting an implementation gap that limits program effectiveness.

### 2.2 Implementation framework

**Fig. 1:**
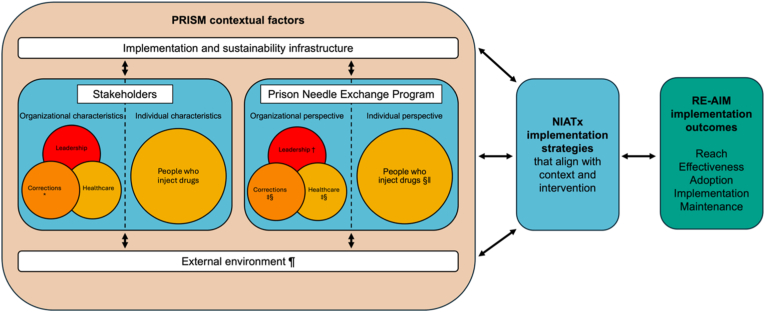
Study implementation logic model. **Notes.** Adapted from https://re-aim.org/learn/prism/. ∗Organisational characteristics will be assessed in staff surveys; organisational perspectives about the PNEP were explored in † Lafferty et al. [29], ‡ Kronfli et al. [30], and § Kronfli et al. [31]; individual perspectives explored in § Kronfli et al. [31] and ‖ Lafferty et al. [32]; and external environment explored in ¶ Kronfli et al. [20].

We used the Practical, Robust Implementation and Sustainability Model (PRISM) [28] to frame our pre-implementation assessment of PNEP within Correctional Service Canada (Fig. 1). PRISM is a process/determinant framework that maps how multilevel context shapes implementation across four domains: 1) organizational and patient characteristics; 2) organizational and patient perspectives of the intervention; 3) external environment (including policy instruments such as the Threat Risk Assessment); and 4) implementation and sustainability infrastructure [33]. PRISM integrates the Reach, Effectiveness, Adoption, Implementation, and Maintenance (RE-AIM) framework [34,35], which provides the structure for evaluating implementation, including participation and clinical/harm reduction measures (Effectiveness), site and staff uptake (Adoption), fidelity (Implementation), and sustainability (Maintenance).

Guided by PRISM, our pre-implementation work using nominal group technique identified site-specific barriers among multi-level stakeholders [30,32]. Correctional officers and healthcare workers identified key barriers for people who inject drugs, including bullying and coercion from peers, fear of being targeted or reprimanded due to drug use from correctional officers, the program's lack of confidentiality/privacy, and the long and complex corrections-mandated Threat Risk Assessment [30]. People who inject drugs in prison similarly reported fear of being targeted by correctional officers or repercussions from correctional officers due to drug use, lack of confidentiality/privacy, and the application process with its strict eligibility criteria as key barriers to participation [32]. Among institutional heads, implementation was framed by structural tensions between harm reduction goals and ‘zero tolerance’ custodial mandates [20,29]. The Threat Risk Assessment was characterized as prioritizing institutional security over public health, and stakeholders proposed transferring PNEP authority entirely to healthcare services and streamlining the approval process [20,31]. Together, these findings informed an *a priori* logic for how contextual features were expected to shape implementation outcomes and will continue to inform process-improvement activities tailored to each prison's context during the trial.

### 2.3 Overall design

This protocol follows the StaRI Statement for implementation studies and incorporates relevant items of the CONSORT Statement extension for stepped wedged cluster randomised trials (Appendices A and B) [36,37]. NEXUS is a type 1 hybrid effectiveness-implementation trial designed to increase uptake of the Canadian PNEP among people who inject drugs in prison by addressing barriers to participation. Type 1 hybrid studies focus primarily on measuring the effectiveness of the intervention, and secondarily explore implementation-related factors [38]. They are suitable when there is existing evidence that the intervention is effective in other settings or populations, supporting its applicability to a new context [39].

All nine federal prisons with an established PNEP at the time of the trial were enrolled, comprising five women's prisons (five multi-level security) and four men's prisons (one minimum security, one medium security, one maximum security, and one multi-level security) (Table 1). Using a stepped-wedge cluster design, each prison will transition from standard of care to NIATx activities over the study period. This design incorporates a six-month baseline period of pre-NIATx data collection which serves as the control condition. We chose a cluster design because NIATx targets organisational-level process improvements that cannot be delivered at the individual level. In a stepped-wedge trial, the effect estimate comparing NIATx to standard of care combines both within-cluster (i.e., outcomes before vs. after implementation within the same prison) and between-cluster (i.e., prisons with the intervention vs. without the intervention at a given time) effects [40]. A key challenge in this design is that outcomes may change over time due to factors unrelated to the intervention [41]. Ideally, the order in which each cluster receives the intervention is randomised to mitigate this potential source of bias.

**Table 1:** Baseline prison needle exchange program participation and prison population size, September 2024-February 2025.

Group	Prison	Province	Security level	Gender	Sep. 2024	Oct. 2024	Nov. 2024	Dec. 2024	Jan. 2025	Feb. 2025
1	Atlantic Institution	New Brunswick	Maximum	Men	17/247 (6.9%)	14/236 (5.9%)	17/224 (7.6%)	17/225 (7.6%)	18/237 (7.6%)	17/225 (7.6%)
	Dorchester Penitentiary	New Brunswick	Multi	Men	10/350 (2.9%)	10/359 (2.8%)	12/362 (3.3%)	14/369 (3.8%)	13/365 (3.6%)	13/368 (3.5%)
	Nova Institution for Women	Nova Scotia	Multi	Women	∼/88 (≤5.7%)	11/86 (12.8%)	9/86 (10.5%)	11/89 (12.4%)	12/93 (12.9%)	17/89 (19.1%)
2	Edmonton Institution for Women	Alberta	Multi	Women	∼/166 (≤3.0%)	∼/169 (≤3.0%)	∼/175 (≤2.9%)	∼/176 (≤2.8%)	∼/177 (≤2.8%)	∼/187 (≤2.7%)
	Grand Valley Institution for Women	Ontario	Multi	Women	∼/220 (≤2.3%)	∼/221 (≤2.3%)	∼/213 (≤2.3%)	∼/228 (≤2.2%)	∼/228 (≤2.2%)	∼/226 (≤2.2%)
	Mission Institution	British Columbia	Medium	Men	14/297 (4.7%)	13/295 (4.4%)	13/289 (4.5%)	13/296 (4.4%)	17/292 (5.8%)	15/294 (5.1%)
3	Fraser Valley Institution for Women	British Columbia	Multi	Women	∼/102 (≤4.9%)	∼/98 (≤5.1%)	∼/104 (≤4.8%)	∼/108 (≤4.6%)	∼/102 (≤4.9%)	∼/104 (≤4.8%)
	Établissement Joliette	Quebec	Multi	Women	∼/103 (≤4.9%)	∼/108 (≤4.6%)	∼/108 (≤4.6%)	∼/106 (≤4.7%)	∼/113 (≤4.4%)	∼/113 (≤4.4%)
	Joyceville Institution	Ontario	Minimum	Men	∼/241 (≤2.1%)	∼/250 (≤2.0%)	∼/260 (≤1.9%)	∼/267 (≤1.9%)	∼/282 (≤1.8%)	∼/278 (≤1.8%)

**Notes.** % of total prison population. ∼ suppressed as count 0≤n ≤ 5; % for these time points are presented as ≤5/total population.

### 2.4 Allocation and sequence generation

**Fig. 2:**
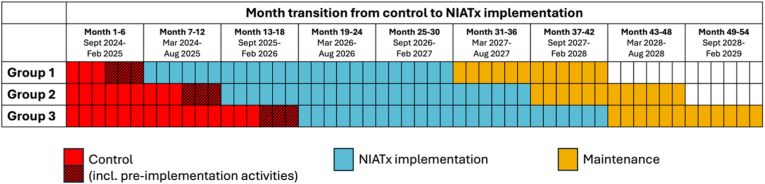
Study timeline.

Although the nine prisons were initially randomised into three sequences, logistical issues required minor adjustments, with one prison per sequence exchanged to accommodate travel and scheduling restrictions. Potential bias from the lack of randomisation will be considered in the interpretation of results. The three sequences are activated at six-month intervals. Each sequence undergoes pre-implementation activities, consisting of NIATx training conducted in the final three months of the six-month baseline control period, followed by 24 months of NIATx implementation, and 12 months of maintenance (Fig. 2). The study commenced in September 2024, with sequence 1 beginning pre-implementation activities in December 2024.

### 2.5 Implementation strategies and tools

Variation in barriers across key stakeholders (i.e., people in prison, staff) and the nine prisons identified in our pre-implementation work led us to select the NIATX model, a blended implementation strategy. NIATx combines practice facilitation, collaborative learning, coaching, rapid cycle change testing (i.e., iterative testing of small changes in practice to refine implementation), and data-driven audit and feedback [42,43], and aligns with the Expert Recommendations for Implementing Change (ERIC) taxonomy [44]. NIATx has been used successfully to improve service delivery and scale up evidence-based interventions, including opioid agonist therapies [[45], [46], [47]]. To our knowledge, NIATx has not previously been applied to harm reduction services in prisons. By extending NIATx into this domain, we adapt a proven blended implementation strategy for organisational change to a carceral context, emphasizing workflow redesign, data-driven decision-making, and multi-level engagement between correctional, clinical, and public health stakeholders, to optimise adoption, implementation, and maintenance.

We will use NIATx to enhance PNEP delivery by addressing site-specific barriers identified in our pre-implementation work. Each prison will establish a three-person change team, including a designated leader and representatives from both healthcare (e.g., nurse) and corrections (e.g., correctional manager). Throughout the study, quarterly meetings with Correctional Service Canada will help ensure ongoing executive sponsorship and be used to monitor the PRISM external environment, including leadership changes, policy shifts, and variations in federal funding, which could influence implementation.

#### 2.5.1 Pre-implementation phase

During this phase, change teams from each sequence of three prisons will participate in a one-day, in-person training session with the study team (including a NIATx coach) on the principles of NIATx. The session will introduce the NIATx bundle of implementation strategies and tools (e.g., rapid cycle testing, walk-throughs, flowcharting) and support teams in identifying potential plan-do-study-act change projects based on site-specific rank-ordered barriers determined with multi-level stakeholders *a priori* [[30], [31], [32]]. Group activities will help solidify these concepts. For example, change teams will practice conducting ‘walk-throughs’ to understand the perspective of potential PNEP participants and use ‘flowcharting’ to map how PNEP activities are currently delivered in their respective prisons. These activities are intended to help change teams reframe barriers as opportunities for process improvement. Teams will be encouraged to use NIATx tools throughout the implementation phase to iteratively identify new or previously unrecognised barriers and generate new ideas for change projects.

#### 2.5.2 Implementation phase

A NIATx coach will lead activities during the 24-month implementation phase. The coach, members of the research team, and each prison change team will meet monthly via videoconference to use NIATx tools to guide process improvement. Each month, the change team will plan and implement a change project using a plan-do-study-act framework, tailored to address site-specific barriers identified through nominal group technique, walk-throughs, and/or flowcharting. For example, some may focus on increasing awareness of the PNEP through discussions with new prison admissions, while others may address the lengthy PNEP application process [30,32]. The role of the NIATx coach in this process is to guide rather than direct, building the capacity of each team to sustain process improvements beyond the implementation phase [43]. Key coaching elements include providing encouragement, positive reinforcement, and troubleshooting to reduce frustration and sustain momentum during the change project process [43]. We anticipate some staff turnover during the implementation phase; when this occurs, departing team members will be replaced by staff from the same department (i.e., health or corrections), who will be trained in the NIATx tools.

Developed in collaboration with Correctional Service Canada, a PNEP ‘data dashboard’ will allow change teams to make data-driven decisions following implementation of their monthly change projects. The dashboard is updated monthly and provided to change team members prior to each coaching call. For example, the number of PNEP applications will be tracked over time to evaluate PNEP uptake, while median time to participation approval will be used to understand timeliness of program access. Change teams will review these data monthly to determine whether to adopt, adapt, or abandon the changes implemented through rapid cycling testing. Over the course of the implementation phase, there will be approximately 24 monthly change projects. A NIATx fidelity form is completed after each coaching call.

Change teams at activated sites will also meet face-to-face every six months for collaborative learning, which aims to facilitate peer-to-peer learning through the sharing of progress, challenges, and promising practices. These collaborative meetings will also support knowledge transfer from the more experienced change teams to the newer teams, aligning with PRISM's Implementation and Sustainability Infrastructure which considers sharing of promising practices a key element.

#### 2.5.3 Maintenance phase

During the 12-month maintenance phase, the NIATx coach will transition facilitation duties to the change leader at each site. The goal is that the internal NIATx coach will oversee continued use of NIATx tools to improve (or sustain) PNEP uptake while continuing to interact with all stakeholder groups.

### 2.6 Participants

All people in the nine prisons who self-report a history of injection drug use are eligible to request participation in the PNEP. Due to ethical and privacy concerns, only aggregate (cluster-period-level) data without individual-level identifiers are available for analysis. Consequently, while this design constitutes an ‘open cohort’ [48], we are unable to track specific individuals over time, so data will be analyzed cross-sectionally despite repeated assessment of some of the same individuals. We anticipate that there will be participant overlap between months, meaning the population composition will remain relatively stable over time.

### 2.7 Study outcomes

The RE-AIM framework will guide evaluation of PNEP effectiveness and implementation outcomes [34] (Table 2).

**Table 2:** Data measures mapped to the RE-AIM framework.

Domain	Definition	Measure	Data source	When measured	Frequency of measurement	Analysis type
Effectiveness	Primary: PNEP uptake	Primary: Count of PNEP participation	Correctional Services Canada	PI, IP, MP	Monthly	Quantitative
	Secondary: a PNEP coverage b Uptake of bloodborne virus services c Uptake of opioid agonist therapies	Secondary: a Count of PNEP kits distributed b Count of people screened, diagnosed, and treated for HIV/HBV/HCV c Count of enrolees on opioid agonist therapies				
Adoption	Increase in the number and/or availability of PNEPs in other Canadian federal prisons	a Number of prisons newly initiating PNEP services b Increased hours of operation of PNEP services	Correctional Services Canada	IP, MP	N/A	Quantitative
Implementation	PNEPs delivered as intended and with fidelity	a Fidelity to NIATx b Individual and organizational factors affecting implementation	b NIATx fidelity checklist and problem indicators subscale c Staff surveys	a IP b IP, MP	Every six months	Quantitative
Maintenance	Extent to which PNEPs are sustained after implementation phase	Maintenance of changes; barriers to maintenance; resource allocation	Nominal group technique	MP	Start and end of MP (∼6-9 months apart)	Mixed

**Abbreviations:** CSC = Correctional Service Canada; PI = pre-implementation phase; IP = implementation phase; MP = maintenance phase.

#### 2.7.1 Effectiveness outcomes

The primary effectiveness outcome in this type 1 hybrid trial is PNEP uptake, defined as a monthly count of PNEP participants divided by the total prison population. Participation is a practical, measurable proxy indicator for PNEP effectiveness, capturing engagement with the program. We acknowledge, however, that high PNEP participation alone does not guarantee that all intended benefits of the program are achieved, particularly in the context of barriers to participation (e.g., confidentiality, stigma). Secondary effectiveness outcomes were selected accordingly and include PNEP coverage (number of needle/syringe kits distributed), opioid agonist therapy uptake (number of people receiving medication), and the numbers of people tested, diagnosed, and treated for bloodborne viruses (HIV, HCV, and hepatitis B virus), each expressed as a monthly count per total prison population. Data will be extracted by Correctional Service Canada at monthly intervals.

#### 2.7.2 Implementation outcomes

Adoption will be documented as (1) the number of new federal prisons that introduce PNEP after the initiation of our study, and (2) expansion of PNEP operating hours, both of which reflect increased PNEP access. These metrics will be monitored through conversations with Correctional Service Canada leadership.

Fidelity will be monitored after every coaching call during the implementation period using an adapted NIATx fidelity checklist. This includes eight items (possible score: 0-3) that relate to structure and process (Appendix C). Structural items capture the role of leadership in NIATx, while process items capture how the change team uses the plan-do-study-act rapid cycle testing activities to improve PNEP implementation. A fidelity score of ≥80% (i.e., ≥19/24) will be considered ‘high’. A five-item problem indicators subscale (Appendix D) will complement the checklist to quantify the magnitude of any issues.

Staff attitudes and organisational characteristics will be captured through surveys administered every six months during all phases of the trial to all healthcare and correctional staff at participating prisons. Surveys include sociodemographic information (e.g., gender, sex, education), staff knowledge and perceptions of PNEP, and several validated brief scales. Work satisfaction and stress will be measured using the ProQOL-5 [49]; COPAN-5 will assess interprofessional collaboration [50]; the Resistance to Change Scale will measure routine seeking, emotional reaction, short term focus and cognitive rigidity [51]; the Social Dominance Scale (SDO_7_) will measure preference for group based hierarchy and inequality [52]; the Attitudes Towards Rehabilitation and Punishment scale will assess the extent to which staff believe that individuals should be punished for their drug use [53]; the Brief Substance Abuse Attitude Survey will capture broader attitudes towards drug use [54]; the Implementation Climate Scale will be used to capture issues relating to PNEP implementation [55]; the Implementation Leadership Scale will assess perspectives on Correctional Service Canada leadership in implementing PNEP [56]; and the Readiness to Change scale will capture staff motivation and organisational climate [57]. Responses will be linked over time using a unique anonymized identifier.

Maintenance will be examined in focus groups using nominal group technique at the beginning and end of the maintenance phase. The same stakeholder groups from preliminary work will participate in each prison: incarcerated individuals (peer advocates and potential and current PNEP users), correctional officers, and healthcare workers. Each group will include 8-10 participants (the ideal size for nominal group technique [58]), with 36 groups in total (4 groups of stakeholders x 9 prisons). Nominal group technique ensures equitable participation in settings where a power imbalance may exist [59]. Focus group prompts will address barriers to sustaining PNEP process changes and strategies required to support ongoing improvements.

### 2.8 Planned statistical analysis

#### 2.8.1 Analysis of the primary outcome

The primary analysis will examine the effect of NIATx on PNEP participation, using a generalised linear mixed model with a negative binomial distribution, comparing the implementation and control periods [41]. The model will include intervention status and time (categorical by month) as fixed effects and a random intercept and period effect (months) at the prison level. Fixed effects represent the group-average structure, with inclusion of time separating the secular trend from the intervention effect [60]. Random effects will account for both within- and between-period intra-cluster correlations. A log offset for prison population size will be included to adjust for differences in prison size and so that results represent the rate of participation, rather than a count of participants. The Kenward-Roger degree-of-freedom correction will be used to account for the small number of clusters. The implementation effect estimate will be expressed as a rate ratio with 95% confidence interval, with the significance level set at α = 0.05.

In a secondary, exploratory non-inferiority analysis of the primary outcome, we will compare the post-implementation (maintenance) period to the late implementation period to examine whether effects are sustained once external facilitation ends. As this is an exploratory analysis and the primary outcome was pre-specified, we will not perform a multiplicity adjustment, with the focus on effect estimates and confidence intervals.

#### 2.8.2 Sensitivity analyses

We will conduct a sensitivity analysis excluding the final three months of the pre-implementation period to reduce the potential for contamination from NIATx training activities.

#### 2.8.3 Power

Power calculations were based on detecting an absolute increase of 5% in PNEP participation, which was considered a realistic and clinically important difference. Estimates were conducted for a fixed sample of nine prisons transitioning over three six-month steps. All model parameters were derived from baseline data from the participating sites (Table 1). Specifically, we incorporated the observed average monthly population of 200 people per prison, a baseline PNEP participation rate of 5%, a within-period intra-cluster correlation coefficient (ICC) of 0.05, and a cluster autocorrelation coefficient (CAC) of 0.95. The correlation structure was modelled with an exponential decay to allow for a different within and between-period ICC. With a two-sided significance level of 0.05, we achieve 95% power to detect a 5% absolute increase (i.e., an increase from 5% to 10%) in PNEP participation. ICC and CAC coefficients were estimated using the ICC Estimation Shiny App [61] and power calculations were performed using the Shiny CRT Calculator [62].

#### 2.8.4 Planned subgroup analyses

We will assess whether the effect of NIATx on PNEP uptake differed by prison gender by including interaction terms between gender and the intervention and period effects. This is relevant because while lifetime injection drug use is higher among women than men in Canadian federal prisons [63], baseline uptake of PNEP in women's prisons is lower. This analysis is exploratory and intended to generate a hypothesis regarding gender as a potential effect modifier.

We will also explore the effects of fidelity to NIATx (measured using the fidelity checklist) and staff characteristics (e.g., readiness to change, attitudes towards people who use drugs) on implementation by including these variables as time-varying predictors in the models. This approach will allow us to assess whether improvements in fidelity or changes in staff attitudes over time are associated with increased effectiveness (i.e., higher PNEP participation), as has been observed in previous studies [64,65].

#### 2.8.5 Secondary effectiveness outcomes

Secondary outcomes (e.g., PNEP coverage, bloodborne virus testing) will be analyzed using the same approach as the primary outcome. Analyses of secondary outcomes will be considered exploratory given that the trial is powered for the primary outcome.

#### 2.8.6 Missing data

We do not anticipate any missing data for the primary or secondary effectiveness outcome measures since they are service-level data extracted by Correctional Service Canada. However, for ethical reasons, counts of ≤5 will be suppressed. We will assume the midpoint (n = 3) where data are suppressed and conduct sensitivity analyses at lower bound (n = 1) and upper bound (n = 5) to assess whether conclusions change.

### 2.9 Ethics and equity

The research protocol was approved by the McGill University Health Centre Research Ethics Board (#2023-9201) and the study is registered on ClinicalTrials.gov (Ref NCT07122219). As data are aggregated by prison, individual consent is not required for people in any of the participating prisons. Confidentiality is protected through data aggregation and suppression of small counts. Staff who complete surveys will provide informed consent, and their responses will be kept confidential.

The carceral setting introduces unique ethical and implementation challenges, most notably the tension between security priorities and health care delivery [20]. To safeguard participants, we will monitor for unintended harms, such as increased surveillance or reprisals related to participation. Mitigation strategies will be informed by regular feedback from staff during coaching calls and ongoing engagement with Correctional Service Canada leadership. We will also explicitly address issues of equity. Uptake of PNEP among women remains low in Canadian federal prisons, with some institutions reporting zero participation; this underrepresentation will be monitored in our analyses to ensure equitable access. Collectively, these procedures are intended to ensure that implementation proceeds in a manner that is ethical and equitable, while upholding prison safety and providing insights that are relevant to policymakers and correctional health decisionmakers.

## 3 Discussion

This pragmatic implementation trial represents the first application of the NIATx process improvement model to a harm reduction intervention within a carceral setting. By adapting NIATx to the rigid hierarchies and security priorities in Canadian federal prisons, this study assesses whether a collaborative, data-driven approach can enhance PNEP effectiveness and sustainability. We aim to determine if structured organisational change methods can overcome entrenched resistance and operational inertia that has historically limited the impact of harm reduction programs in prisons globally. Findings will inform national policy, support global scale-up of PNEPs, and advance implementation science in high-security settings.

Global experience with PNEPs shows highly variable outcomes. While some countries (e.g., Switzerland, Spain, Moldova) have achieved broad reach through diverse distribution models, others (e.g., Germany, Kyrgyzstan) have seen service reductions due to misalignment between health and correctional authorities [13]. This variability underscores the need for adaptive, iterative implementation strategies that can tailor interventions to local context while maintaining core principles of safety, confidentiality, and equity. The blended NIATx model offers this flexibility, emphasizing local leadership, rapid cycle testing, data transparency, team-based learning, and collaborative learning to support the translation of national policy into daily operational practice.

For Canada, where the introduction of PNEPs was litigation-driven rather than health-system led, this trial has immediate policy relevance. It provides a structured pathway for Correctional Service Canada to scale up PNEPs consistent with the *2024-2030 Sexually Transmitted and Bloodborne Infection Action Plan*. Furthermore, it establishes a framework for embedding across the federal prison harm reduction portfolio, including opioid agonist therapies. By focusing on process redesign and stakeholder co-ownership, NIATx addresses barriers through better use of existing resources, offering a model that is both cost-efficient and sustainable.

Beyond Canada, this work advances implementation science by demonstrating the feasibility of blended implementation strategies within highly regulated carceral environments. It offers a roadmap to generate the robust evidence required to support World Health Organization and United Nations Office on Drugs and Crime recommendations for scaling harm reduction amidst the competing priorities of secure settings. Amid ongoing challenges of incarceration and injection-related infections, embedding process improvement approaches such as NIATx in carceral settings helps bridge the persistent gap between policy and practice, supporting more equitable, data-informed care for people who inject drugs in prison.

Prisons are highly controlled settings with well-documented challenges associated with conducting research [66]. To undertake this study, we compromised on some elements of an ‘ideal’ implementation trial. Ethical considerations necessitated measurement of effectiveness outcomes at the prison level, rather than at the individual level, and logistical factors precluded randomisation of the intervention. While conducting research under sub-optimal conditions is not unusual in carceral settings, we acknowledge the limitations of these compromises. The lack of individual-level data has several implications: (1) we do not know the number of people in prisons who inject drugs and therefore would benefit from PNEPs, meaning that we cannot accurately measure ‘reach’ within the RE-AIM framework, which aims to evaluate the proportion and representativeness of people who participate; (2) we cannot adjust analyses for important confounders, including age and risk behaviours; (3) we cannot conduct subgroup analyses, other than by gender, which restricts our ability to examine potential equity issues (e.g., access by Indigenous ethnicity); (4) we cannot differentiate between participants' security level at multi-level prisons, which is important considering concerns among people classified as minimum-security, where PNEP participation may jeopardize their release [29]; (5) analysis at the prison-level reduces statistical power because the effective sample size is limited to the number of prisons, rather than the number of individuals; and (6) because the data are restricted to monthly aggregate counts with a somewhat stable population composition, we cannot distinguish between new PNEP initiation and continued use by the same individuals over time. Finally, non-randomisation of the prisons introduces potential bias in rollout order, which makes distinguishing between intervention effects and secular trends more challenging. It also results in a quasi-experimental rather than experimental design, which requires stronger assumptions in the analysis and limits causal inference. Despite these constraints, this study is positioned to generate robust evidence on the effectiveness and implementation of PNEPs in Canadian federal prisons and is the first of its kind globally.

## 4 Conclusion

This study aims to improve the implementation of Canadian PNEPs, thereby increasing program uptake and reducing bloodborne virus transmission. By systematically examining the contextual factors that influence PNEP delivery and effectiveness, this study will support the sustainability of existing programs and their scale up within Canada and beyond.

## Funding

This study was funded by the Canadian Institutes of Health (CIHR) HIV/AIDS Research Initiative (190374, 185725, and 195710). OP is supported by a post-doctoral fellowship award from the CIHR Pan-Canadian Network for HIV and STBBI Clinical Trials Research Network (CTN+). FLA is supported by several research grants funded by the National Institute on Drug Abuse (R01 DA029910, R01 DA054851) in the United States. BH is supported by an Investigator Grant from the Australian National Medical Research Council (2026968). NK is supported by a career award from the Fonds de Recherche Québec – Santé (FRQ-S; Junior 2).

## CRediT authorship contribution statement

**Olivia Price:** Methodology, Visualization, Writing – original draft, Writing – review & editing. **Frederick L. Altice:** Conceptualization, Funding acquisition, Methodology, Supervision, Writing – review & editing. **Lynn M. Madden:** Methodology, Writing – review & editing. **Monica Taljaard:** Methodology, Supervision, Writing – review & editing. **Mark Stoové:** Funding acquisition, Methodology, Writing – review & editing. **Behzad Hajarizadeh:** Funding acquisition, Methodology, Writing – review & editing. **Lise Lafferty:** Funding acquisition, Methodology, Writing – review & editing. **Nadine Kronfli:** Conceptualization, Funding acquisition, Methodology, Supervision, Writing – review & editing.

## Declaration of competing interest

OP, FLA, MT, BH and LL report no competing interests. MS has received investigator-initiated research funding from Gilead Sciences and AbbVie and consultant fees from Gilead Sciences for activities unrelated to this work. NK has received investigator-initiated research funding from ViiV Healthcare, Abbvie, and Gilead, and speaker fees from Abbvie, none of which involve this research.

## Figures and Tables

**Table:** 

Checklist item		Reported on page #	Implementation Strategy	Reported on page #	Intervention
			“Implementation strategy” refers to how the intervention was implemented		“Intervention” refers to the healthcare or public health intervention that is being implemented.
**Title and abstract**					
Title	1	1	Identification as an implementation study, and description of the methodology in the title and/or keywords		
Abstract	2	4	Identification as an implementation study, including a description of the implementation strategy to be tested, the evidence-based intervention being implemented, and defining the key implementation and health outcomes.		
**Introduction**					
Introduction	3	5	Description of the problem, challenge or deficiency in healthcare or public health that the intervention being implemented aims to address.		
Rationale	4	5	The scientific background and rationale for the implementation strategy (including any underpinning theory/framework/model, how it is expected to achieve its effects and any pilot work).	5	The scientific background and rationale for the intervention being implemented (including evidence about its effectiveness and how it is expected to achieve its effects).
Aims and objectives	5	6	The aims of the study, differentiating between implementation objectives and any intervention objectives.		
**Methods: description**					
Design	6	7-8	The design and key features of the evaluation, (cross referencing to any appropriate methodology reporting standards) and any changes to study protocol, with reasons		
Context	7	6	The context in which the intervention was implemented. (Consider social, economic, policy, healthcare, organisational barriers and facilitators that might influence implementation elsewhere).		
Targeted ‘sites’	8	8	The characteristics of the targeted ‘site(s)’ (e.g., locations/personnel/resources etc.) for implementation and any eligibility criteria.	11	The population targeted by the intervention and any eligibility criteria.
Description	9	9-11	A description of the implementation strategy	6-7	A description of the intervention
Sub-groups	10	n/a	Any sub-groups recruited for additional research tasks, and/or nested studies are described		
**Methods: evaluation**					
Outcomes	11	12-13	Defined pre-specified primary and other outcome(s) of the implementation strategy, and how they were assessed. Document any pre-determined targets	11-12	Defined pre-specified primary and other outcome(s) of the intervention (if assessed), and how they were assessed. Document any pre-determined targets
Process evaluation	12	12-13	Process evaluation objectives and outcomes related to the mechanism by which the strategy is expected to work		
Economic evaluation	13	n/a	Methods for resource use, costs, economic outcomes and analysis for the implementation strategy	n/a	Methods for resource use, costs, economic outcomes and analysis for the intervention
Sample size	14	14	Rationale for sample sizes (including sample size calculations, budgetary constraints, practical considerations, data saturation, as appropriate)		
Analysis	15	13-15	Methods of analysis (with reasons for that choice)		
Sub-group analyses	16	14	Any *a priori* sub-group analyses (e.g. between different sites in a multicentre study, different clinical or demographic populations), and sub-groups recruited to specific nested research tasks		
**General**					
Statements	27	2, 15-16	Include statement(s) on regulatory approvals (including, as appropriate, ethical approval, confidential use of routine data, governance approval), trial/study registration (availability of protocol), funding and conflicts of interest		

**Notes.** Adapted from Ref. [37] with checklist items relating to the results and discussion removed for the purpose of this protocol paper.

**Table:** 

Topic	Item no	Checklist item	Page no
Title and abstract			
	1a	Identification as a stepped wedge cluster randomised trial in the title.	1
	1b	Structured summary of trial design, methods, results, and conclusions (see separate SW-CRT checklist for abstracts).	3
Introduction			
Background and objectives	2a	Scientific background. Rationale for using a cluster design and rationale for using a stepped wedge design.	5, 7-8
	2b	Specific objectives or hypotheses.	6
Methods			
Trial design	3a	Description and diagram of trial design including definition of cluster, number of sequences, number of clusters randomised to each sequence, number of periods, duration of time between each step, and whether the participants assessed in different periods are the same people, different people, or a mixture.	7-8
	3b	Important changes to methods after trial commencement (such as eligibility criteria), with reasons.	n/a
Participants	4a	Eligibility criteria for clusters and participants.	6, 11
	4b	Settings and locations where the data were collected.	6
Interventions	5	The intervention and control conditions with sufficient details to allow replication, including whether the intervention was maintained or repeated, and whether it was delivered at the cluster level, the individual participant level, or both.	9-11
Outcomes	6a	Completely defined prespecified primary and secondary outcome measures, including how and when they were assessed.	11-13
	6b	Any changes to trial outcomes after the trial commenced, with reasons.	n/a
Sample size	7a	How sample size was determined. Method of calculation and relevant parameters with sufficient detail so the calculation can be replicated. Assumptions made about correlations between outcomes of participants from the same cluster. (see separate checklist for SW-CRT sample size items).	14
	7b	When applicable, explanation of any interim analyses and stopping guidelines.	n/a
Randomisation			
Sequence generation	8a	Method used to generate the random allocation to the sequences of treatments.	8
	8b	Type of randomisation; details of any constrained randomisation or stratification, if used.	n/a
Allocation concealment mechanism	9	Specification that allocation was based on clusters; description of any methods used to conceal the allocation from the clusters until after recruitment.	n/a
Implementation	10a	Who generated the randomisation schedule, who enrolled clusters, and who assigned clusters to sequences.	n/a
	10b	Mechanism by which individual participants were included in clusters for the purposes of the trial (such as complete enumeration, random sampling; continuous recruitment or ascertainment; or recruitment at a fixed point in time), including who recruited or identified participants.	n/a
	10c	Whether, from whom and when consent was sought and for what; whether this differed between treatment conditions.	n/a
Blinding	11a	If done, who was blinded after assignment to sequences (eg, cluster level participants, individual level participants, those assessing outcomes) and how.	n/a
	11b	If relevant, description of the similarity of treatments.	n/a
Statistical methods	12a	Statistical methods used to compare treatment conditions for primary and secondary outcomes including how time effects, clustering and repeated measures were taken into account.	13-15
	12b	Methods for additional analyses, such as subgroup analyses, sensitivity analyses, and adjusted analyses.	13-15
Other information			
Registration	23	Registration number and name of trial registry.	15
Protocol	24	Where the full trial protocol can be accessed, if available.	n/a
Funding	25	Sources of funding and other support (such as supply of drugs), and the role of funders.	2
Research ethics review	26	Whether the study was approved by a research ethics committee, with identification of the review committee(s). Justification for any waiver or modification of informed consent requirements.	15

**Notes.** Adapted from Ref. [36] with checklist items relating to the results and discussion removed for the purpose of this protocol paper.

**Table:** 

Fidelity Measure
**I. Structural Components** (Ratings 0 = Never; 1 = Sometimes; 2 = Usually 3 = Always)
1. Change Team has had one consistent leader (or satisfactory transitions from one leader to another)
2. Study objective guides the team (Increase # of PNEP participants)
3. Change Team has enough support to execute change
**II. Process Components** (Ratings 0 = Never; 1 = Sometimes; 2 = Usually 3 = Always)
1. (Plan) Team considers new ideas as a group
2. (Do) Changes are executed one at a time
3. (Study) Data are recorded & reviewed by the team
4. (Act) At the end of each cycle, the team decides to adopt, adapt, or abandon change
5. Change projects are amenable to rapid cycle testing (i.e., 4-6 weeks)

## Data Availability

Data will be made available on request.

## Appendix D Problem indicators subscale

Ratings: 0 = major problem; 1 = minor problem; 2 = not a problem.

Q1. Meetings too frequently cancelled or poorly attended to get the work done.
Q2. Team's work or schedule was interrupted/discontinued.
Q3. Facility Sponsor doesn't show adequate interest in/support for the Change Team's efforts.
Q4. Team membership turns over too frequently to have enough team understanding of plan-do-study-act.
Q5. Team leader is ineffective at moving team through cycle.
